# Cognitive functions and jugular venous reflux in severe mitral regurgitation: A pilot study

**DOI:** 10.1371/journal.pone.0207832

**Published:** 2019-02-22

**Authors:** Shih-Hsien Sung, Ching-Wei Lee, Pei-Ning Wang, Hsiang-Ying Lee, Chen-Huan Chen, Chih-Ping Chung

**Affiliations:** 1 Division of Cardiology, Department of Medicine, Taipei Veterans General Hospital, Taipei, Taiwan; 2 Cardiovascular Research Center, National Yang Ming University, Taipei, Taiwan; 3 Institute of Public Health, National Yang Ming University, Taipei, Taiwan; 4 School of Medicine, National Yang Ming University, Taipei, Taiwan; 5 Department of Neurology, Neurological Institute, Taipei Veterans General Hospital, Taipei, Taiwan; 6 Brain Research Center, National Yang Ming University, Taipei, Taiwan; 7 Department of Medical Education, Taipei Veterans General Hospital, Taipei, Taiwan; Scuola Superiore Sant'Anna, ITALY

## Abstract

Cardiac diseases with elevated central venous pressure have higher frequency of jugular venous reflux (JVR), which is associated with decreased cerebral blood flow and white matter hyperintensities. Whether patients with severe mitral-regurgitation (SMR) have poorer cognitive functions and whether JVR is involved were determined in this pilot study. Patients with SMR and age/sex-matched controls were prospectively recruited. Neuropsychological tests such as global cognitive (Mini-Mental State Examination, MMSE), verbal memory, executive, and visuospatial domains were performed. Cardiac parameters by cardiac catheterisation and echocardiography, and the frequency of JVR by colour-coded duplex ultrasonography were obtained. Forty patients with SMR and 40 controls (71.1±12.2, 38–89 years; 75% men) were included. Compared with the controls, patients with SMR had lower scores in all neuropsychological tests but only MMSE and visuospatial test scores were statistically significant after adjusting for age, sex, and educational level. We further adjusted for cardiovascular risk factors; the significance remained in the visuospatial test but diminished in MMSE. Multivariate linear regression analyses adjusted for age, sex, and educational level showed that JVR combined with high right-atrial-pressure (RAP > 50th-percentile, 12 mmHg) was significantly associated with poorer performances in both MMSE [right JVR: B coefficient(95% confidence interval,*p*) = -2.83(-5.46–0.20, 0.036); left JVR: -2.77(-5.52–0.02, 0.048)] and visuospatial test [right JVR: -4.52(-8.89–0.16, 0.043); left JVR: -4.56(-8.81–0.30, 0.037)], with significances that remained after further adjusting for cardiovascular risk factors. Our pilot results suggest that retrogradely-transmitted venous pressure might be involved in the mechanisms mediating the relationship between cardiac diseases and brain functions.

## Introduction

Cerebral venous drainage impairment with elevated venous pressure would decrease cerebral blood flow (CBF), damage the blood–brain barrier (BBB), and lead to brain dysfunctions [1,2]. Internal jugular vein (IJV) is the largest extracranial vein for cerebral venous drainage [1]. Jugular venous reflux (JVR) indicates a retrograde flow in IJV, which usually occurs when the reversed pressure gradient was elevated beyond the capacity of the IJV valves [3]. We previously showed that during Valsalva’s manoeuvre (VM), people with JVR would decrease CBF and dilate retinal venules more than ones without JVR [4,5]. In addition, JVR has been found to be associated with white matter hyperintensities (WMH) in the elderly people [6]. These results indicate that JVR might influence CBF, cerebral microvessels, and brain tissues via elevated venous pressure retrogradely transmitted into the cerebral venous system.

Continuous or repeated elevated venous pressure proximal to IJV might result in wear and tear of the IJV valves and lead to valvular incompetence [3,7,8]. Indeed, certain cardiac diseases with increased central venous pressure such as heart failure or valvular heart disease have a higher frequency of JVR [7,8]. Recently, the number of studies that have reported cognitive impairment in patients with cardiac diseases has been increasing [9]. However, whether cerebral venous return status is a factor involved in the relationship between cardiac diseases and cognitive function remains to be elucidated. The present study compared neuropsychological performances between patients with severe mitral valve regurgitation (SMR) and age- and sex-matched normal controls. Patients with SMR will encounter pulmonary venous hypertension at the beginning, followed by combined pre and post-capillary pulmonary hypertension during disease progression [10,11]. The right ventricular pressure, as well as the right atrial pressure (RAP), increased thereafter, which may lead to IJV valvular incompetence and compromise the cerebral venous return [10,11]. We hypothesised that patients with SMR have poorer cognitive functions and the presence of JVR and/or elevated RAP might be associated with cognitive impairment in these patients.

## Materials and methods

### Study population

Patients with SMR, referred for surgical intervention in a tertiary medical centre, were eligible for this study. Every patient underwent transthoracic and transoesophageal echocardiography and cardiac catheterisation to confirm the diagnosis and to evaluate the feasibility for surgery. Patients who had disease durations of 1 year or longer from the initial diagnosis to the time of catheterisation and echocardiography were included. Among the eligible patients, those who met the following criteria were excluded from this analysis: (1) had concomitant severe aortic valve disease, mitral stenosis, acute coronary syndrome, or pericardial disease; (2) had unstable haemodynamics, or New York Heart Association functional class IV symptoms; (3) had existing neurological diseases, such as stroke, brain tumour, dementia, or other neurodegenerative diseases; and (4) had significant stenosis (>50%) over the cervical internal carotid and vertebral arteries using neck duplex sonography. Cardiac diseases other than mitral valvular disease were excluded because they might have different mechanisms and effects on the cognitive functions. A total of 40 individuals were included based on these criteria. We also recruited 40 age- and sex-matched normal controls from outpatients who visited our neurological clinics. These normal controls had no cardiac, neurological, or malignant medical histories.

Cardiovascular risk factors were either measured or assessed through self-report. The presence of hypertension was determined by a self-report of current antihypertensive medication prescription or by a measurement of either systolic BP of ≥140 mmHg or diastolic BP ≥90 mmHg [12]. Diabetes mellitus (DM) was defined by either a self-report of current DM medication or a measurement of haemoglobin A1c (HgbA1c) of ≥6.5% [13]. Chronic kidney disease (CKD) was defined according to an estimated glomerular filtration rate (eGFR) of ≤60 mL/min/1.73 m^2^ [14]. The design of this study was reviewed and approved by the institutional review board of Taipei Veterans General Hospital. All participants had signed informed consent paper document before included in the present study.

### Cardiac catheterisation

Cardiac catheterisation was performed in all patients with SMR using a percutaneous approach via the radial artery for coronary angiogram and right IJV for right heart catheterisation. Data of mean pulmonary artery wedge pressure (PAWP), pulmonary artery pressure (PAP), right ventricular pressure (RVP), RAP, mixed venous oxygen saturation (SvO_2_), and cardiac output were obtained. Cardiac output was then divided by body surface area (BSA) to obtain the cardiac index.

### Echocardiography

A comprehensive two-dimensional, M-mode, and Doppler echocardiogram was performed by a skilled echocardiographer using commercially available echocardiographic devices (Philip IE33, Andover, MA, USA) following a standardised protocol. The severity of mitral regurgitation was evaluated according to the effective regurgitant orifice, which was calculated by analysing the proximal isovelocity hemispheric surface area (PISA) of the convergence on the atrial side. According to the AHA/ACC guideline, and an effective regurgitant orifice of ≥ 0.4 cm^2^ was referred as SMR [15]. The severity of tricuspid regurgitation (TR) was evaluated with qualitative and semi-quantitative parameters, while severe TR is defined when the jet width of vena contracta is more than 7mm. To determine mild or moderate TR, we assessed the tricuspid valve morphology, color flow of TR jet, and continuous wave Doppler signal of TR jet according to the guideline [15].Both left and right heart structures and functions were obtained, including left ventricular end-diastolic and end-systolic dimension, left atrial dimension, left atrial volume, and estimated right ventricular systolic pressure. Left ventricular ejection fraction (LVEF) was obtained using biplane Simpson’s method, and left ventricular mass was measured using the area–length method. The peak trans-mitral filling velocity at early diastole (E), septal mitral annulus moving velocity at early diastole (e′), and E/e′ratio were also obtained. All parameters were measured in triplicate and averaged according to the guideline of the American Society of Echocardiography. Decompensated heart failure was defined as reduced LVEF (<35%) with chronic clinical symptoms (≥6 months) of New York Heart Association functional class III–IV.

### Colour-coded duplex ultrasonography: JVR determination

Neck colour-coded duplex sonography was performed in all patients with SMR using a 7-MHz linear transducer (iU22; Philips, New York, NY, USA) by the same technician who was blinded to subjects’ characteristics. On examination, subjects were in a head-straight, flat supine position after a quiet 10-min rest. The IJV was initially insonated longitudinally and thoroughly from the proximal part of the neck base rostrally to the distal part of the submandibular level to detect any possible spontaneous JVR at baseline. Then, the VM was performed by forcible expiration by the subject via the mouth into a flexible rubber tube connected to a manometer. Subjects were asked to reach the 40 mmHg Valsalva pressure and maintain it for at least 10 s. During the VM, the distal margin window of the colour signal was placed at the tip of the flow divider of the internal carotid artery. The coloured box was adjusted to include the entire lumen of the IJV; if retrograde colour appeared in the centre of the lumen, the retrograde flow would then be confirmed by Doppler spectrum. JVR was determined when the retrograde-flow colour in the centre of the lumen and the Doppler-flow waveform demonstrated reversed flow for >0.5 s spontaneously or/and during VM [3–6].

Routine cervical arterial examination including examination of internal carotid and vertebral arteries was also performed in all patients with SMR.

### Cognitive function assessment

All patients with SMR and normal controls underwent a face-to-face neuropsychological examination carried out by trained interviewers. In addition to the global cognitive performance, which was examined using the Mini-Mental State Examination (MMSE), three different cognitive domains (verbal memory, visuospatial function, and executive function) were assessed using extensive neuropsychological tests as follows:

- Verbal memory: delayed (10 min) free recall in the Chinese Version of the Verbal Learning Test (CVVLT) [16].
- Visuospatial function: the copy of the Taylor complex figure test [17].
- Executive function: digit backward test [18].

### Statistical analysis

Analyses were performed using SPSS software (v22.0, IBM, Armonk, NY, USA). All continuous variables are described as mean ± standard deviation (SD) and discrete variables as percentages. Comparisons of case and control were made using non-parametric Mann–Whitney tests. When appropriate, chi-square (χ2) or Fisher’s exact tests were performed for categorical variables. Univariate and multivariate linear regression analyses of neuropsychological test scores as the dependent variable were performed. Adjusted confounding factors were age, sex, educational level, cardiovascular risk factors (hypertension, DM, hyperlipidaemia, cigarette smoking, alcohol consumption, and CKD) and the status of TR (mild, moderate and severe).

To test our postulation that cerebral venous return status might be involved in the relationship between cognitive impairment and SMR, we analysed the hemodynamic parameters that may affect cerebral venous return, e.g., the RAP and presence of JVR, as independent variables individually. We also used the 50th percentile of the mean RAP, with 12 mmHg as a cut-off point. Three kinds of binary category variables, (1) RAP ≥ and <12 mmHg, (2) the presence or absence of JVR, and (3) the presence or absence of combined JVR and high RAP (≥12 mmHg), were individually analysed as independent variables. Furthermore, since decreased cardiac output is commonly postulated as a contributor to cognitive impairment in cardiac diseases, we also put cardiac index and LVEF into analyses.

## Results

Table 1 shows the demographics and neuropsychological test scores of 40 patients with SMR and 40 age-/sex-matched control. The patient group had higher frequency of cardiovascular risk factors, except cigarette smoking; however, the difference was statistically significant only in the frequency of CKD. Among the patients with SMR, 10 (25%) had decompensated heart failure, 13 (32.5%) had moderate TR and 6 (15.0%) had severe TR.

**Table 1:** Comparisons of demographics and cognitive functions between patients with severe mitral regurgitation and normal controls.

	SMR (n = 40)	Control (n = 40)	*P*
Age, years, mean (SD, range)	71.1 (12.2, 38–89)	71.1 (12.2, 38–89)	-
Sex, man, n (%)	30 (75.0)	30 (75.0)	-
Education, years, mean (SD)	10.6 (4.8)	10.3 (4.9)	0.903
Hypertension, n (%)	22 (55.0)	15 (37.5)	0.178
Diabetes mellitus, n (%)	8 (20.0)	5 (12.5)	0.546
Hyperlipidemia, n (%)	10 (25.0)	3 (7.5)	0.066
Cigarette smoking, n (%)	12 (30.0)	13 (32.5)	1.000
Chronic kidney disease, n (%)	20 (50.0)	5 (5.0)	<0.001
**Age, sex, education adjusted**			
MMSE, mean (SD)	26.1 (5.1)	27.8 (2.4)	0.020
Verbal memory: CVVLT 10 min, mean (SD)	6.4 (3.0)	6.9 (1.8)	0.387
Executive function: digit backward test, mean (SD)	5.4 (2.7)	6.4 (3.0)	0.081
Visuospatial function: the Taylor complex figure test, mean (SD)	29.8 (6.9)	31.9 (4.0)	0.040

SMR = severe mitral regurgitation; CVVLT = Chinese Version of the Verbal Learning Test.

The patient group had lower scores in all neuropsychological tests compared with control group, but only statistically significant in MMSE and Taylor complex figure test and borderline significant in digit backward test after adjusting for age, sex, and educational level. We further adjusted for cardiovascular risk factors, with significance remaining in the Taylor complex figure test (*p* = 0.046) but lower in MMSE (*p* = 0.058).

**Fig 1:**
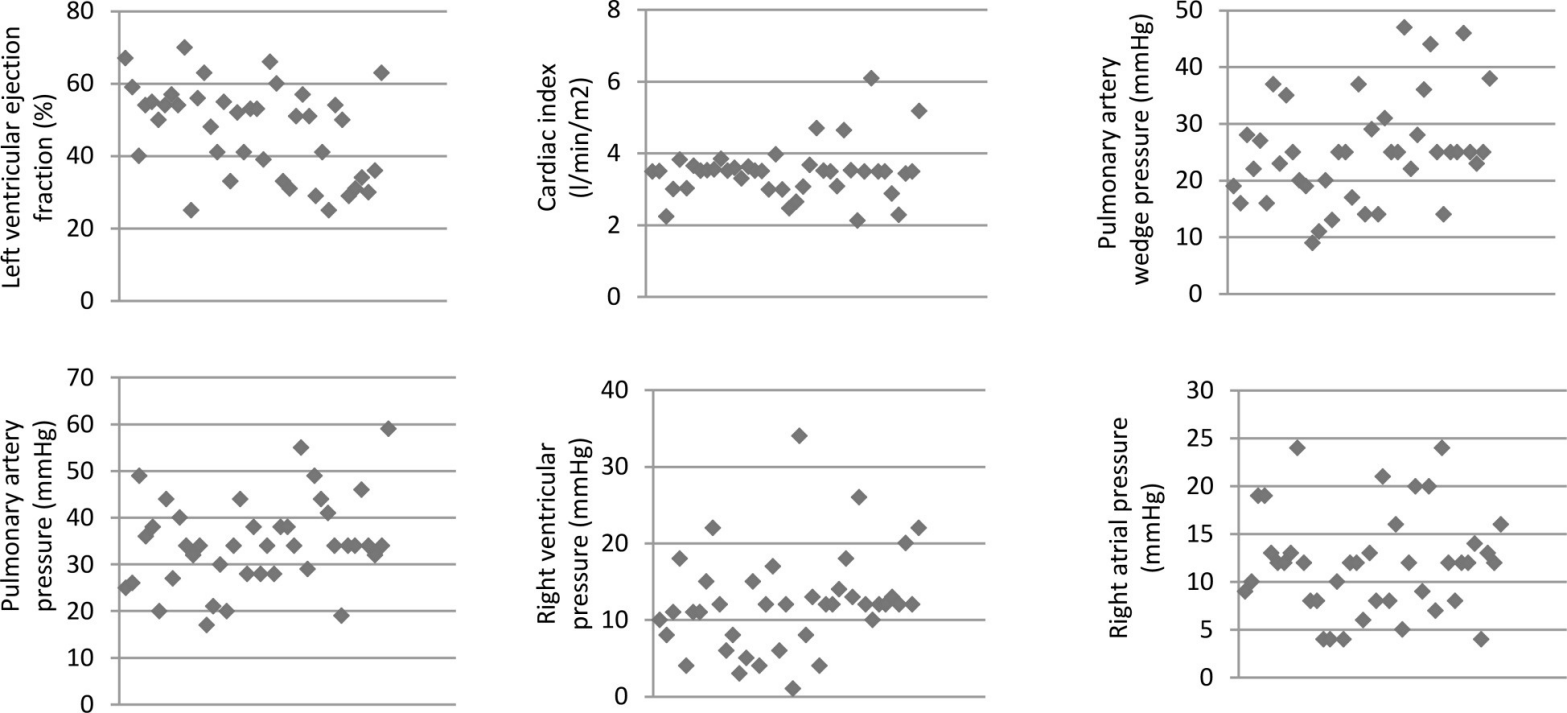
Distributions of hemodynamic measurements in patients with severe mitral regurgitation.

Table 2 and Fig 1 shows the hemodynamic parameters measured by cardiac catheterization and the frequency of JVR detected by color-coded duplex ultrasonography in SMR patients. An elevated mean RAP and high frequency of JVR were observed in patients with SMR.

**Table 2:** Hemodynamic parameters in patients with severe mitral regurgitation.

LV ejection fraction, %, (SD, range)	47.3 (12.6, 25–70)
Cardiac index, l/min/m^2^, (SD, range)	3.5 (1.0, 2.1–6.1)
PAWP, mmHg, (SD, range)	25.2 (10.4, 9–47)
PAP, mmHg, (SD, range)	34.7 (10.7, 17–59)
RVP, mmHg, (SD, range)	12.3 (7.3, 1–34)
RAP, mmHg, (SD, range)	11.9 (5.9, 4–24)
Right JVR, n (%)	20 (50.0)
Left JVR, n (%)	22 (55.0)

LV = left ventricle; PAWP = pulmonary artery wedge pressure; PAP = pulmonary artery pressure; RVP = right ventricular pressure; RAP = right atrial pressure; JVR = jugular venous reflux.

**Fig 2:**
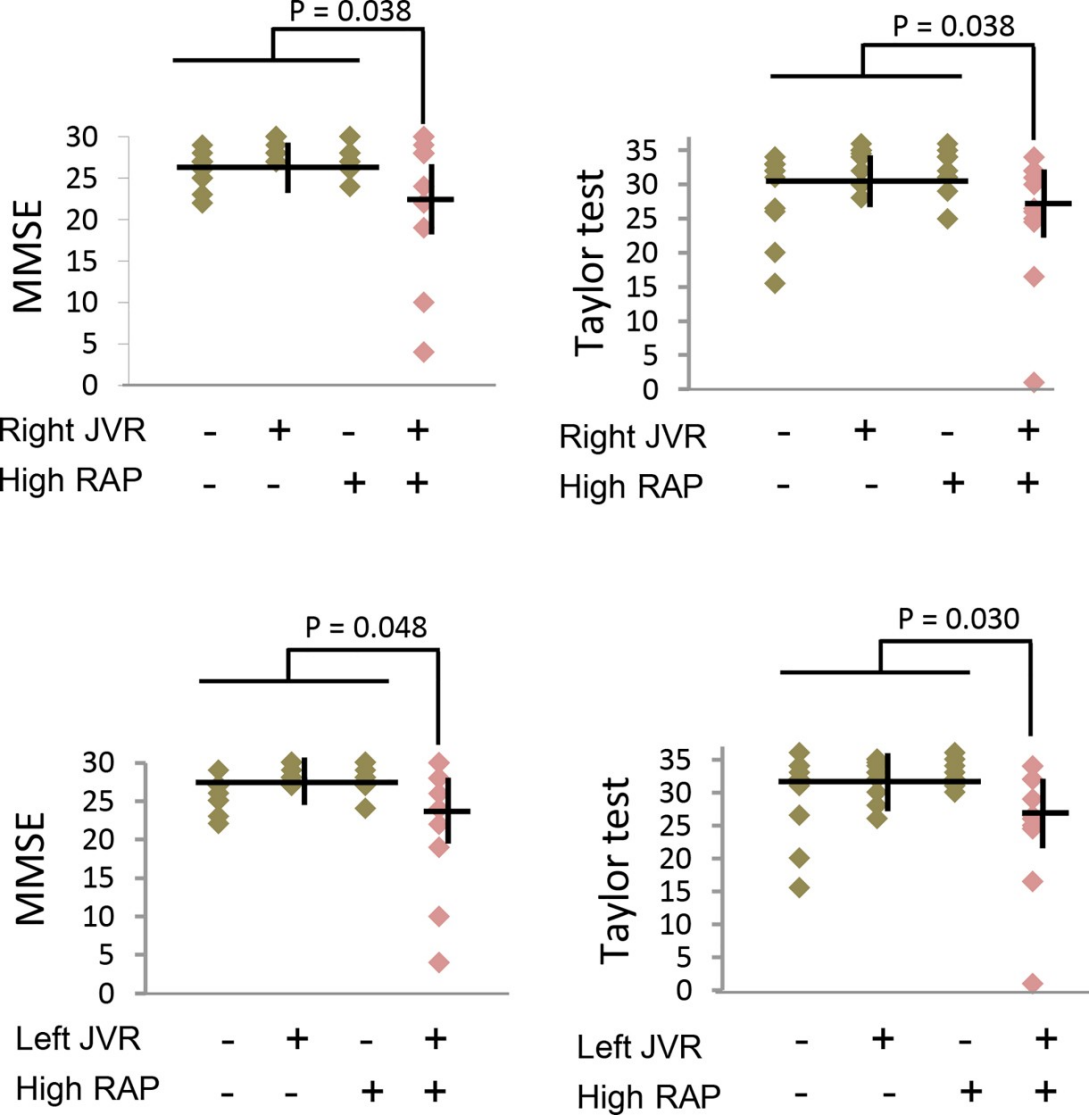
Cognitive functions in four groups of patients with severe mitral valve regurgitation classified according to the presence or absence of jugular venous reflux and high right atrial pressure.

We then performed multivariate analyses to test which haemodynamic parameter was associated with poorer cognitive domains, e.g., MMSE and the Taylor complex figure test, in patients with SMR (Table 3). Multivariate analyses adjusted for age, sex, and educational level showed that cardiac index, LVEF, mean RAP, high mean RAP (≥12 mmHg), or presence of right or left JVR were not associated with the MMSE and Taylor complex figure test scores. However, JVR combined with high mean RAP was significantly associated with poorer performances both in MMSE and Taylor complex figure test. The significances remained after further adjusting for cardiovascular risk factors and the status of TR. We also divided patients into four groups according to the presence or absence of JVR and high mean RAP. Fig 2 shows the mean scores of MMSE and Taylor complex figure test of the four groups. Cognitive functions in patients with isolated JVR or high mean RAP were not poorer than those with the absence of JVR and high mean RAP; however, JVR combined with high mean RAP had the lowest scores in both MMSE and Taylor complex figure test among the four groups. Multivariate analyses showed that patients in the group of JVR combined with high mean RAP had significantly poorer performances in both MMSE and Taylor complex figure test compared with those in the other three groups.

**Table 3:** Associations of cardiac parameters with cognitive functions in patients with severe mitral regurgitation.

	**Mini-Mental Status Examination**					
	B (95% CI)	*P*^a^	B (95% CI)	*P*^b^	B (95% CI)	*P*^c^
LVEF	0.04 (-0.08–0.16)	0.532				
Cardiac index	-1.03 (-2.77–0.72)	0.232				
RAP	-0.05 (-0.27–0.17)	0.640				
RAP > 12 mmHg	-1.33 (-3.79–1.13)	0.278				
Right JVR	-1.14 (-4.49–2.21)	0.494				
Left JVR	-0.72 (-4.04–2.61)	0.664				
Right JVR & RAP > 12 mmHg	-2.83 (-5.46 - -0.20)	0.036	-3.05 (-5.92 - -0.19)	0.038	-3.06 (-5.99 - -0.13)	0.041
Left JVR & RAP > 12 mmHg	-2.77 (-5.52 - -0.02)	0.048	-2.96 (-5.89 - -0.02)	0.048	-2.98 (-5.99 - -0.12)	0.048
	**Visuospatial function: the Taylor complex figure test**					
	B (95% CI)	*P*^a^	B (95% CI)	*P*^b^	B (95% CI)	*P*^c^
LVEF	0.10 (-0.05–0.28)	0.237				
Cardiac index	0.10 (-2.58–2.79)	0.936				
RAP	-0.08 (-0.44–0.28)	0.648				
RAP > 12 mmHg	-0.92 (-4.93–3.10)	0.642				
Right JVR	-1.51 (-6.11–3.09)	0.507				
Left JVR	-3.05 (-7.47–1.37)	0.169				
Right JVR & RAP > 12 mmHg	-4.52 (-8.89 - -0.16)	0.043	-4.93 (-9.56 - -0.30)	0.038	-5.09 (-9.73 - -0.44)	0.033
Left JVR & RAP > 12 mmHg	-4.56 (-8.81 - -0.30)	0.037	-4.96 (-9.40 - -0.52)	0.030	-5.06 (-9.48 - -0.63)	0.027

LVEF = left ventricle ejection fraction; RAP = right atrial pressure; JVR = jugular venous reflux; B = B coefficient; CI = confidence interval.

^a^adjusted for age, sex and education years.

^b^adjusted for age, sex, education years and cardiovascular risk factors (hypertension, diabetes mellitus, hyperlipidemia, cigarette smoking, alcohol consumption, and chronic kidney disease).

^c^adjusted for age, sex, education years, cardiovascular risk factors (hypertension, diabetes mellitus, hyperlipidemia, cigarette smoking, alcohol consumption, and chronic kidney disease) and the status of TR (mild, moderate and severe).

## Discussion

The main findings were that patients with SMR had (1) poorer global cognitive (MMSE) and visuospatial (the Taylor figure test) functions compared with those in normal controls and (2) JVR combined with high RAP was associated with these cognitive impairments. The present pilot study was of limited scope, having a small sample size. Therefore, our findings should be interpreted with caution and no firm conclusions should be made regarding the general applicability of our findings at this moment in time. Future studies including larger sample size are needed to confirm the validity of our findings.

We previously reported that the prevalence of JVR in the general population (16–89 years old) is approximately 18–36% on the right side and 6–29% on the left side [19]. The present study showed a high frequency of JVR (50–55%) in patients with SMR. Chronic SMR with a continuous or repeated elevated central venous pressure might wear and tear the IJV valves and lead to valvular incompetence. This postulation is supported by a high RAP found in our SMR patients and the other studies showing a higher frequency of JVR in heart failure or tricuspid valve disease which have elevated central venous via elevated RAP.

Although retrogradely transmitted venous pressure by JVR has been shown to reach the cerebral venous system and influence CBF [3–6], the extent of induced cerebral venous hypertension is milder than that of the other conditions, such as dural arteriovenous fistula (DAVF) [20–22]. Therefore, compared with diffuse cerebral white matter hyperintensities (WMH) caused by DAVF [20–22], JVR is only associated with WMH over caudal brain (occipital, thalamus, and infratentorial brain regions) in which venous drainage pathway is closer to IJV [6]. In addition, age is needed to enhance JVR-related brain insults; JVR is associated with the severity of WMH only in people aged ≥75 years [6]. The present study had similar observations. Merely the presence of JVR was not associated with SMR-related cognitive impairment; nevertheless, with the additional high RAP, JVR was associated with poorer cognitive performances, global cognitive (MMSE), and visuospatial (the Taylor figure test) functions in patients with SMR (Fig 2). Our results lead to the postulation that high RAP related to heart failure has limited influence on the brain if IJV valves are competent; JVR with high RAP can cause brain dysfunction via retrogradely transmitted venous pressure only when IJV valves are incompetent.

Several studies on brain–heart axis have emerged, and they have shown a relationship between cognitive impairment and cardiac diseases [9]. Most studies were focusing on heart failure and little on the effect of mitral valve disease on cognitive functions [9,23]. Our results showed that compared with age- and sex-matched normal controls, patients with SMR had poorer global cognitive performance (MMSE) and visuospatial function (Taylor figure test) after adjusting with educational level. The diminished significance of association in MMSE after adjusting for cardiovascular risk factors suggests that more prevalent cardiovascular risk factors such as hypertension, DM, and CKD might be contributors to poorer global cognitive function in SMR. Notably, the anatomic correlations of visuospatial function impairment, significantly and independently associated with SMR, include the occipital lobe, which is one of the JVR-susceptible regions [6]. This result also supports our postulated mechanism mediating the cognitive impairment in SMR.

Cerebral circulation includes artery supply and venous drainage. Both of them are responsible for adequate CBF and brain metabolic homeostasis [2,24]. Recently, several studies have indicated that, in addition to maintaining adequate CBF and BBB function, waste and lymphatic clearance are dependent on cerebral venous drainage [25–27]. However, a greater proportion of studies are focusing on the arterial side, e.g., cardiac output, when evaluating the relationship between the circulation (heart) and the brain [9]. Results of the present study indicate a role of the venous side in the impact of cardiac disease on brain dysfunction. We did not find associations between parameters reflecting the arterial side, such as cardiac index and LVEF and cognitive functions in patients with SMR. Our results are consistent with those of a recent study [28]. They investigated the association between various cardiac haemodynamic parameters and the volume of WMH in chronic valvular heart disease such as mitral valve regurgitation (43.1% of the study population) and found that RAP is associated with WMH. In their results, instead of cardiac index, LVEF, and other cardiac hemodynamic parameters, only the mean RAP is significantly, independently, and linearly associated with the WMH volume. However, they did not investigate the neurological functions and competence of IJV valves in those patients. The role of JVR on these valvular heart disease-related WMHs and whether WMH is associated with cognitive impairment as shown in our study were unclear.

We noticed that CI and LVEF in our patients were relatively higher than patients with heart failure. The study population was composed of patients with degenerative and functional MR, while patients with degenerative MR generally have preserved LV functions and cardiac index. We also acknowledged that cardiac output could be over-estimated using either thermos-dilution or indirect Fick’s method. There was also one case who had mean PCWP less than 10mmHg (9mmHg). This patient was a case of severe MR along with severe TR. Even though the PCWP and RAP were low, the presence of prominent V wave confirmed the diagnosis of severe MR and TR. The other possible mechanism for low PCWP was over-diuresis status.

Given a relatively small sample size and to avoid inadequate size in any classified group (as independent variables), we choose 50^th^ percentile of mean RAP as the cut-off point to test our postulation whether a higher RAP with JVR was associated with cognitive impairment in patients with severe MR. This value would need further studies with a larger sample size to validate before applying to clinical settings.

The present study has limitations. The study sample size was relatively small. Heterogeneous profiles of cardiovascular risk factors and hemodynamic parameters might decrease statistical power or confound the current results. Furthermore, the cross-sectional study setting could not establish a causal relationship. A larger and longitudinal study is necessary to validate our postulation. In addition, more investigated tools such as brain imaging are needed to further evaluate the underlying mechanisms between the cerebral venous drainage impairment and cognitive abnormalities in SMR.

## Conclusions

Patients with SMR had poorer cognitive function, particularly in the visuospatial domain, and JVR combined with high RAP was associated with poorer visuospatial function in these patients. The results suggest that retrogradely transmitted venous pressure but not low cardiac output might be involved in the mechanisms mediating the relationship between valvular heart disease and brain functions. In addition to management for decreasing RAP, IJV valve repair might be a potential treatment option for cardiac disease-related brain dysfunctions.
